# Agglomeration: when folded proteins clump together

**DOI:** 10.1007/s12551-023-01172-4

**Published:** 2023-12-15

**Authors:** M. L. Romero-Romero, H. Garcia-Seisdedos

**Affiliations:** 1 Max Planck Institute of Molecular Cell Biology and Genetics, Dresden, Germany; 2 Center for Systems Biology, Dresden, Germany; 3 Department of Structural and Molecular Biology, Institut de Biologia Molecular de Barcelona (IBMB-CSIC), Barcelona, Spain

**Keywords:** Protein-protein interactions, Protein assembly, Protein aggregation, Supramolecular assemblies, Biomolecular condensates

## Abstract

Protein self-association is a widespread phenomenon that results in the formation of multimeric protein structures with critical roles in cellular processes. Protein self-association can lead to finite protein complexes or open-ended, and potentially, infinite structures. This review explores the concept of protein agglomeration, a process that results from the infinite self-assembly of folded proteins. We highlight its differences from other better-described processes with similar macroscopic features, such as aggregation and liquid-liquid phase separation. We review the sequence, structural, and biophysical factors influencing protein agglomeration. Lastly, we briefly discuss the implications of agglomeration in evolution, disease, and aging. Overall, this review highlights the need to study protein agglomeration for a better understanding of cellular processes.

## Introduction

Protein self-association is a prevalent phenomenon across organisms (Goodsell and Olson 2000; Levy and Teichmann 2013), primarily driven by non-covalent interactions. Such associations give rise to multimeric protein structures that play crucial roles in diverse cellular processes, including signal transduction (Park et al. 1999), allosteric regulation (Monod et al. 1965), and protein homeostasis (Sigler et al. 1998).

These associations can take finite forms, resulting in closed assemblies (Ahnert et al. 2015; Levy et al. 2008), or infinite forms, leading to the formation of open-ended, often insoluble supramolecular structures (Dobson 2003; Garcia-Seisdedos et al. 2019; Yeates 2017). Traditionally, apart from cytoskeleton proteins, like actin, tubulin, and intermediate filaments (Alberts et al. 2002), infinite assemblies have been linked to misfolding and aggregation (Garcia-Seisdedos et al. 2019). When proteins misfold, hydrophobic residues become exposed to the solvent, leading to non-specific interactions with other misfolded proteins, resulting in the formation of amorphous (Boatz et al. 2017) or amyloid-like aggregates (Dobson 2003). Protein aggregation is typically irreversible, toxic to cells, and frequently associated with diseases (Chiti and Dobson 2006; Dobson 2003; Fink 1998).

However, recent research challenges the notion that infinite assemblies necessarily arise from misfolding. Point mutations, as observed in hemoglobin S in sickle cell disease (Eaton and Hofrichter 1990; Harrington et al. 1997) and certain mutant forms of γD-crystallin in cataracts (Boatz et al. 2017), can induce folded proteins to self-assemble into infinite assemblies (Empereur-Mot et al. 2019; Garcia-Seisdedos et al. 2017; Garcia Seisdedos et al. 2022). Changes in the intracellular environment can also trigger metabolic enzymes to assemble into filaments (Munder et al. 2016; Narayanaswamy et al. 2009; Park and Horton 2019; Petrovska et al. 2014). These assemblies appear to serve various functions, such as regulating enzymatic activity (Kim et al. 2010; Stoddard et al. 2020), preventing degradation (Petrovska et al. 2014), promoting cellular dormancy (Montrose et al. 2020), and, in some cases, being associated with aging (Paukštytė et al. 2023).

Crucially, in contrast to protein aggregates, these cases share the common characteristics of maintaining folded protein structures within the assemblies and displaying reversibility (Garcia-Seisdedos et al. 2019). Thus, to distinguish this phenomenon from aggregation, we propose the term “agglomeration” (Garcia-Seisdedos et al. 2019).

In this review, we define the term agglomeration and highlight its differences from other better-described processes with similar macroscopic features, such as aggregation and liquid-liquid phase separation. We review the structural and biophysical factors influencing protein agglomeration and briefly give an overview of the implications of agglomeration in evolution and disease.

## What is protein agglomeration?

*In vitro*, protein precipitation is familiar to every molecular biologist with some experience in protein purification. Under certain conditions, such as mutations or changes in buffer composition, proteins can become insoluble, which can complicate subsequent biochemical studies. Protein precipitation has traditionally been attributed to misfolding and aggregation, a process driven by a loss of protein stability (Chiti and Dobson 2017). At the same time, techniques like salt-driven precipitation, using agents such as ammonium sulfate, have long been employed in protein purification (Duong-Ly and Gabelli 2014; Wingfield 2001). It is generally accepted that these techniques do not induce protein misfolding; instead, they reduce the solubility of folded proteins, which can be easily resolubilized using standard buffers (Duong-Ly and Gabelli 2014; Wingfield 2001). These seemingly contradictory phenomena underscore the diverse origins of protein insolubility, challenging the default association of this process with aggregation.

*In vivo*, i.e., at the cellular level, the widespread use of fluorescent protein tags and advances in fluorescent and electron microscopy techniques, have enabled the visualization and characterization of various biomolecular condensates. These structures include membrane-less bodies like stress granules (Cherkasov et al. 2013; Wallace et al. 2015), germ granules (Brangwynne et al. 2009), and p-bodies (Decker and Parker 2012), as well as protein filaments (Alberti et al. 2009; Petrovska et al. 2014; Stoddard et al. 2020).

Although protein precipitates and biomolecular condensates share similar *in vitro* and *in vivo* macroscopic features, they result from fundamentally different biophysical processes, such as aggregation, liquid-liquid phase separation (LLPS), and agglomeration. While protein aggregation and LLPS are well-described processes that rationalize how proteins can demix from the solvent in different liquid or solid phases (Hyman et al. 2014; Sontag et al. 2014), agglomeration has received far less attention and has often been confused with aggregation, emphasizing the need to differentiate these phenomena for a comprehensive understanding of their cellular functions.

### Agglomeration

We define *agglomeration* as the infinite assembly of folded proteins (Garcia-Seisdedos et al. 2019). Agglomerates exhibit several distinctive features: (i) In agglomeration, the constituents of the assemblies, referred to as protomers, are folded proteins. Therefore, in agglomeration, protomers acquire new intermolecular contacts, driving their self-assembly. Importantly, they retain all of their native contacts and maintain their native structure. (ii) Agglomerates are open-ended and potentially infinite assemblies. This means that as long as the concentration of free protomers is sufficiently high, agglomerates can continue to grow in size. In some cases, they can form micron-scale protein structures within cells (Garcia-Seisdedos et al. 2017; Garcia Seisdedos et al. 2022). (iii) Agglomeration is a reversible process. Meaning that protomers within the assemblies can dissociate from them. This dissociation can occur due to conformational changes (Eaton and Hofrichter 1990), dilution (Garcia-Seisdedos et al. 2017), or environmental changes, such as variations in pH or macromolecular crowding (Munder et al. 2016; Petrovska et al. 2014). Importantly, this dissociation happens without the need for a third protein partner, such as disaggregases or other components of the proteostatic machinery.

### Differences between agglomeration and aggregation

**Fig. 1:**
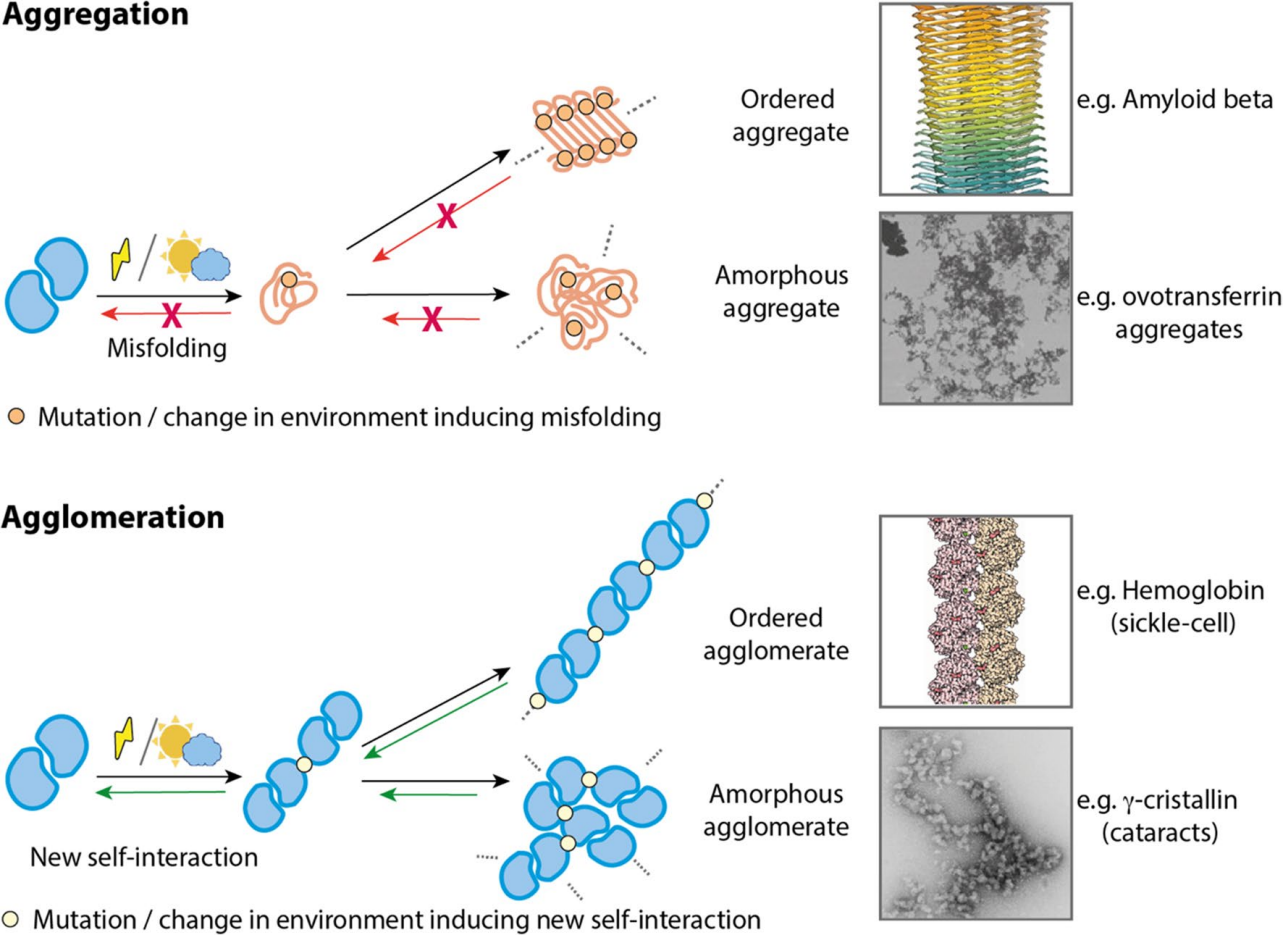
**Contraposition of agglomeration and aggregation concepts**. Aggregation is mediated by protein misfolding, which can be driven by destabilizing mutations or by changes in the environment (e.g., temperature, pH). Misfolded proteins can assemble into ordered structures such as amyloid beta or amorphous aggregates such as the ovotransferrin aggregates (images reproduced from Gremer et al. (2017) and Constantinescu et al. (2017)). In contrast, agglomeration is mediated by the creation of a new protein interaction between two well-folded proteins. Such interaction can be induced by a mutation, as in sickle cell disease, or by changes in the environment. Agglomerates can be ordered, as the filaments of hemoglobin s in sickle cell disease, or amorphous, as in some types of cataracts (images reproduced from Harrington et al. (1997) and (Boatz et al. 2017))

Unlike agglomeration, aggregation is driven by misfolding (Chiti and Dobson 2006). Protein constituents of aggregates lose part of their native contacts which are replaced by new ones with other misfolded proteins, resulting in their assembly into high-order structures (Fig. 1). Aggregates, like agglomerates, are also potentially infinite structures. However, unlike agglomerates, aggregates are not reversible. They are often low-energy hyper-stable structures requiring energy-consuming cellular components like disaggregases and other chaperones (Bukau et al. 2006; Rosenzweig et al. 2019) for dissociation. There are some exceptions though, as some protein aggregates have been observed to dissolve and refold upon dilution (Iadanza et al. 2018). However, the extremely slow kinetics of dissolution and refolding make this process generally irrelevant to biological timescales (Iadanza et al. 2018). Lastly, while aggregation is generally deleterious for fitness (Geiler-Samerotte et al. 2011), agglomeration does not necessarily pose a threat to the cell, as supported by the physiological roles of many agglomerates formed by natural proteins (Nüske et al. 2020; Petrovska et al. 2014; Prouteau et al. 2017; Stoddard et al. 2020). It is worth mentioning that prions constitute a special group of aggregates. Prions are misfolded proteins that self-propagate of their folded counterparts. Although functional prions are common, and whether they are misfolded or low-complexity protein conformations are debatable, they are usually prevented by the presence of high kinetic barriers (Franzmann and Alberti 2019)

While agglomeration and aggregation are distinct, instances like domain swapping blur their boundaries. In this scenario, supramolecular assemblies are composed of interlaced well-folded protomers that retain most of their native contacts (Guo and Eisenberg 2006). Some examples of domain swapping are RNase A (Guo and Eisenberg 2006; López-Alonso et al. 2010) and bacterial pili (Busch and Waksman 2012). However, the unfolding of the protomers precedes domain swapping (López-Alonso et al. 2010). In that respect, we could consider domain swapping midway between aggregation and agglomeration.

### Differences between agglomeration and LLPS-driven condensates

A widespread class of membrane-less bodies consists of condensates formed through liquid-liquid phase separation (LLPS). These LLPS condensates exhibit similarities with agglomerates, as both structures involve native proteins, are reversible, and form open assemblies (Alberti and Hyman 2021). However, there are notable differences between these two processes: (i) LLPS is often mediated or associated with intrinsically disordered domains (Banani et al. 2017; Heidenreich et al. 2020; Pak et al. 2016; Wright and Dyson 2015), resulting in liquid-like properties (Banani et al. 2017; Gomes and Shorter 2019; Hyman et al. 2014). In contrast, agglomerates consist of folded protein units that assemble into either disordered (Boatz et al. 2017; Pande et al. 2005) or ordered solid-like structures, such as filaments (Garcia-Seisdedos et al. 2017; Marini et al. 2020), lattices (Gonen et al. 2015), and crystals (Lanci et al. 2012). (ii) Agglomerates are considered homogeneous structures in which the identity of the constituent protomers is well-defined (Garcia-Seisdedos et al. 2019). On the other hand, LLPS condensates are highly heterogeneous in composition, often containing multiple protein types and nucleic acids (Banani et al. 2017).

## Factors linked to agglomeration

Protein solubility is defined as the concentration of protein in a saturated solution that is in equilibrium with a solid phase under specific conditions (Arakawa and Timasheff 1985; Kramer et al. 2012; Scatchard et al. 1943). Solubility is influenced by a range of factors, including both intrinsic and extrinsic elements.

In the context of agglomeration, intrinsic factors encompass aspects such as the symmetry of the quaternary protein structure and the surface chemistry of the protein (Empereur-Mot et al. 2019; Garcia-Seisdedos et al. 2017, 2019; Garcia Seisdedos et al. 2022). Extrinsic factors, when considering cellular processes, include variables like intracellular protein concentration and the physicochemical properties of the cellular environment (Munder et al. 2016; Petrovska et al. 2014). In this section, we will analyze these factors.

### Symmetry of quaternary structure

**Fig. 2:**
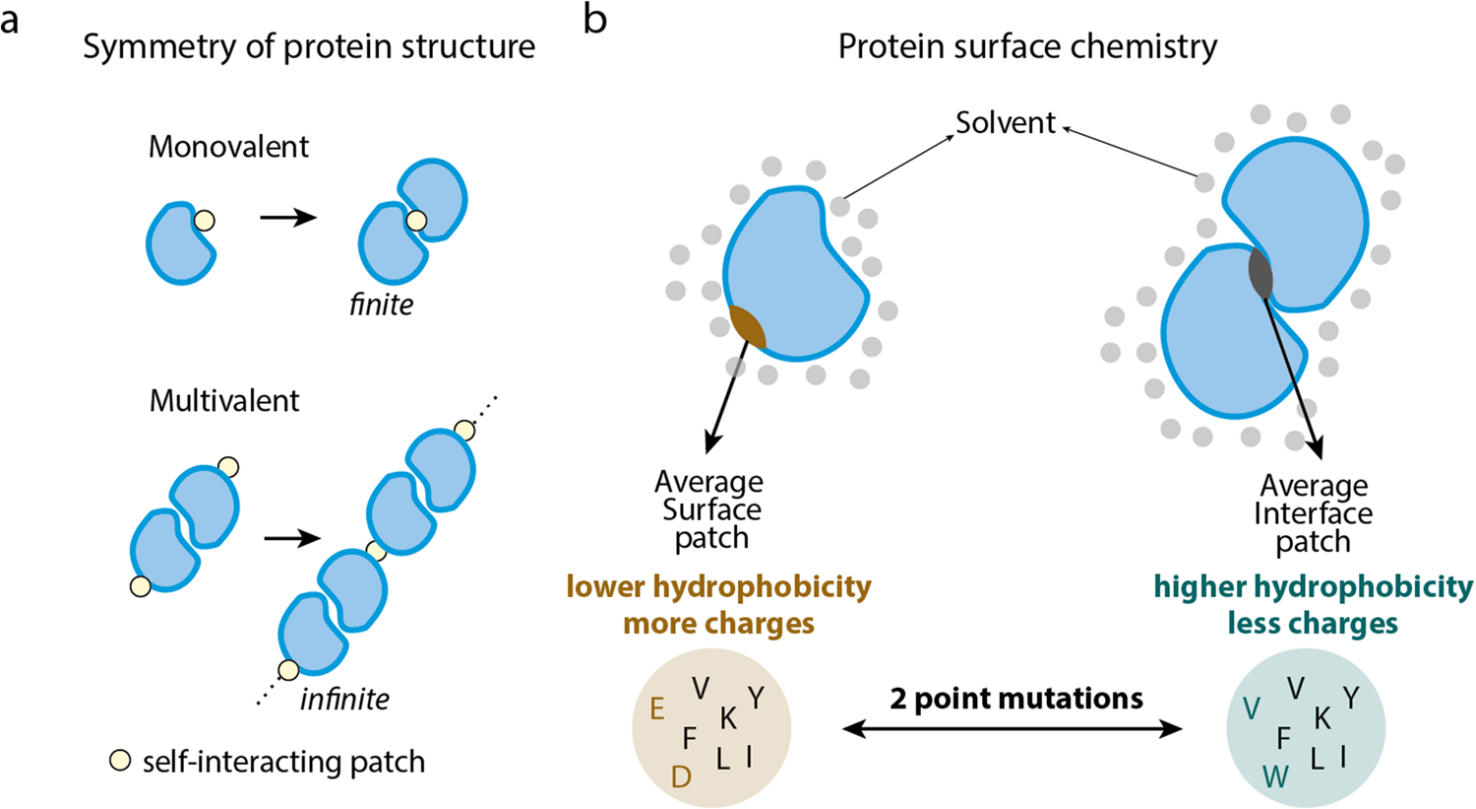
**Intrinsic factors linked to agglomeration**. **a** Symmetry of protein structure is intimately tied to agglomeration as it confers multivalence. A monomeric protein gaining a self-interacting patch forms a dimer, whereas a self-interacting patch repeated on opposite sides of a symmetric protein may result in an infinite assembly. **b** On average, only two substitutions are required to shift the average composition of a surface patch into an interface patch and *vice versa* (Levy 2010)

Agglomeration of proteins is intimately tied to the symmetry of their quaternary structure. Symmetric complexes are prevalent in agglomerates, and specifically, dihedral homomers represent more than 60% of proteins known to form agglomerates (Garcia-Seisdedos et al. 2019). Dihedral homomers are those protein complexes that form in such a way that they exhibit two orthogonal axes of rotational symmetry, with one of them being twofold. This implies that the complex can be rotated 180° around one of the axes and still maintain its original configuration. The overrepresentation of dihedral homomers in agglomerates likely responds to several factors:

1. Structural and sequence similarity increases the interaction propensity of proteins (Lukatsky et al. 2007; Wright et al. 2005). Partly because the probability of finding a self-matching pattern between two identical surfaces is higher compared with the probability of a pattern match between two different surfaces (Lukatsky et al. 2007). Additionally, when two identical surfaces interact, each amino acid residue is repeated twice, increasing its contribution — either favorable or unfavorable — to the free energy association (Lukatsky et al. 2006; Wales 1998). Thus, in a context where interfaces are created randomly and stable ones are selected, interfaces involving two identical protein surfaces have higher chances of selection than those composed of distinct ones (André et al. 2008; Lukatsky et al. 2007; Schulz 2010). This phenomenon could explain the anomalously higher fraction of homodimers relative to heterodimers observed across proteomes (Ispolatov et al. 2005).
2. The repetition of subunits within symmetric complexes introduces multivalence. This property is central in polymer and supramolecular chemistry. Multivalent molecules naturally assemble into polymers or oligomers when mixed, decreasing molecules’ solubility due to entropy-driven effects (Flory 1953), which might promote their phase separation (Banani et al. 2017). Multivalency has been harnessed for the design of distinct protein agglomerates such as protein fibers (Garcia-Seisdedos et al. 2017; Garcia Seisdedos et al. 2022; Grueninger et al. 2008; Padilla et al. 2001), lattices (Gonen et al. 2015; Padilla et al. 2001; Suzuki et al. 2016), and crystals (Lanci et al. 2012; Padilla et al. 2001). Furthermore, the interaction between two symmetric complexes potentially implies multiple binding sites, which increases the strength and specificity of interactions in a cooperative manner (Curk et al. 2017; Fasting et al. 2012; Romero Romero et al. 2018).
3. New self-interactions among symmetric complexes often result in open-ended assemblies (Empereur-Mot et al. 2019; Garcia-Seisdedos et al. 2017, 2019; Yeates 2017). When a new self-interaction is created on a monomer or a cyclic complex, it will likely result in a dimer or a finite complex. Whereas when occurring on a complex with dihedral symmetry, the new self-interaction will necessarily trigger an open-ended, and potentially infinite, assembly (Fig. 2a).

### Protein surface chemistry

For a new protein assembly to take place, a new protein interaction must be created. Theoretical work showed that the chemical composition of a protein interface is very similar to the protein surface. In fact, only two amino-acid substitutions are enough to turn the average surface patch into the average interface patch in terms of chemical composition (Fig. 2b) (Levy 2010). This work suggests that point mutations often lead to the formation of new protein assemblies. If these assemblies are open-ended, they can potentially give rise to new agglomerates.

Several experimental works support this theory. In sickle cell disease, a point mutation at the surface of human hemoglobin that substitutes a charged residue with a hydrophobic one (mutation E6V) results in the formation of a new interface between hemoglobins. As human hemoglobin is symmetric, the acquired interaction results in the formation of fibers (Eaton and Hofrichter 1990). In recent work, Garcia-Seisdedos et al. examined whether point mutations designed to increase the hydrophobicity of the surface of 12 dihedral homomers would trigger similar results. They showed that all 12 homomers underwent agglomeration, confirming that minimal increments in the hydrophobicity of proteins’ surfaces frequently drive new self-interactions (Garcia-Seisdedos et al. 2017).

In a different work, the same authors performed random mutations at the surface of two dihedral homomers. The results indicated that two different mutational pathways lead the homomers to agglomerate. One route involved increasing proteins’ surface hydrophobicity, as we previously demonstrated (Garcia-Seisdedos et al. 2017). A second route implies the sole removal of charged residues from the surface. Notably, replacing a few charged residues for alanines in eight dihedral homomers confirmed that the sole neutralization of surfaces commonly drives agglomeration and intracellular changes in the localization of proteins (Garcia Seisdedos et al. 2022).

The burial of hydrophobic residues at protein interfaces is energetically favorable due to desolvation effects (Chothia and Janin 1975; Garcia-Seisdedos et al. 2012; Romero et al. 2022). It is thus expected that mutations introducing hydrophobic residues are prone to trigger new self-interactions. Indeed, hydrophobicity in protein surfaces can be a burden that is often counterbalanced by negative design, where specific chemical properties evolve to minimize the risk of undesirable protein association events (Doye et al. 2004). Taking into account that protein interactions are concentration-dependent, highly expressed proteins are at particular risk, and consistently, they generally exhibit less hydrophobic interfaces to prevent promiscuous interactions (Levy et al. 2012). Negative design can operate not only on specific proteins but also on sensitive sites on their surface to reduce undesirable self-assembly events (Empereur-Mot et al. 2019; Garcia-Seisdedos et al. 2017; Pechmann et al. 2009)

Even more surprising is that simply removing charges from protein surfaces can create new interfaces (Garcia Seisdedos et al. 2022). This implies the presence of interaction-prone patches on protein surfaces, which are normally shielded by charged residues to prevent their association. Why such sticky patches exist in some protein surfaces is not fully understood. One possibility is that these sticky patches might facilitate the conditional assembly of proteins under specific cellular environments, such as changes in pH (Garcia Seisdedos et al. 2022). A decrease in pH could neutralize gatekeeper charges on protein surfaces, thereby enabling protein self-association. Such a phenomenon is suggested to regulate some enzymes’ oligomerization (Andréll et al. 2009) and could plausibly explain the pH-dependent agglomeration of many metabolic enzymes (Munder et al. 2016).

Disulfide bridges between cysteines and metal coordination can also trigger intermolecular association of protomers and promote agglomeration *in vitro* (Suzuki et al. 2016). In this work, Suzuki and co-workers minimally redesigned the surface of a C4 homomer by introducing cysteines and histidines. Thus, oxidation of the cysteines and coordination of Zn^2+^ and Cu^2+^ ions by histidines resulted in the formation of two-dimensional lattices. A similar strategy was followed to create one-, two-, and three-dimensional protein arrays through the coordination of Zn^2+^ ions also by means of surface histidine residues (Brodin et al. 2012). Previously, Lawson and co-workers designed a ferritin crystal by coordination of Ca^2+^ ions through glutamine and aspartic residues (Lawson et al. 1991). In addition to proteinogenic amino acids such as histidines, glutamines, aspartic, and glutamates (Song et al. 2014), metal coordination can also be achieved by non-natural and chemically modified amino acids in the design of protein agglomerates (Tavenor et al. 2017; Yang and Song 2019).

Other kinds of interactions, such as Met S-Aromatics, have the potential to create protein agglomerates (Valley et al. 2012).

### Protein concentration

**Fig. 3:**
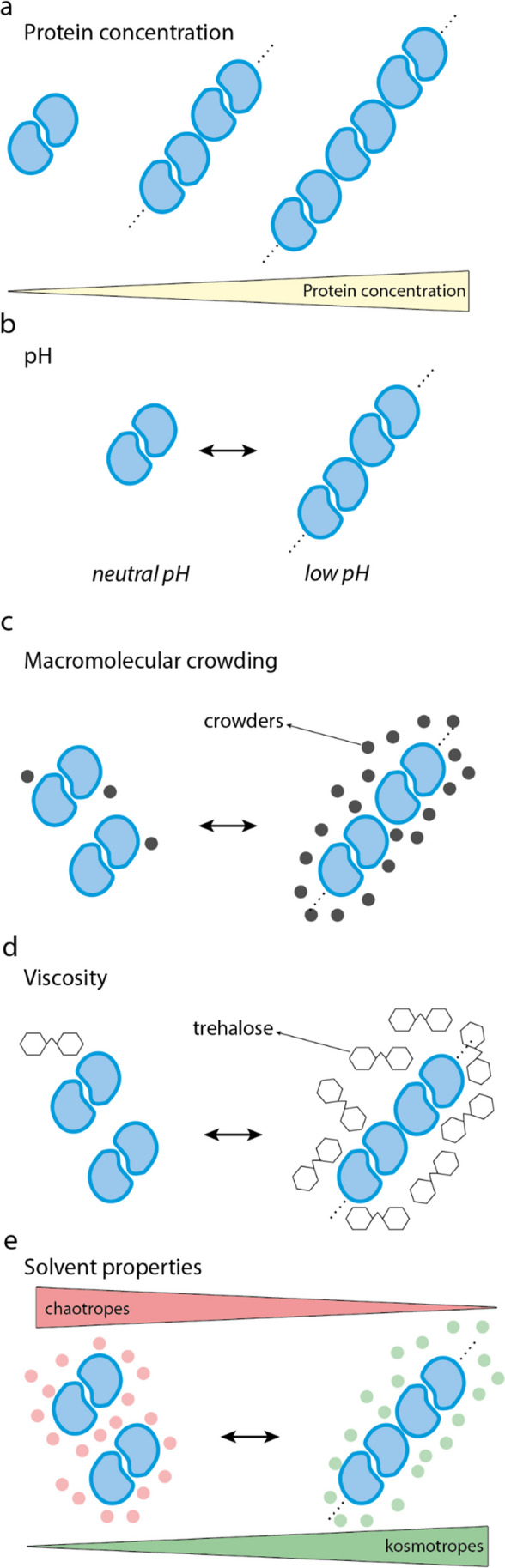
**Extrinsic factors linked to agglomeration**. Protein concentration, pH, macromolecular crowding, viscosity, and co-solutes are extrinsic factors modulating protein agglomeration

Protein binding is a process that depends on the concentration of its constituents. In principle, every protein could agglomerate provided that its concentration is high enough. Consequently, proteins that are highly expressed in the cells are at particular risk of protein agglomeration (Fig. 3a). Indeed, sickle cell disease happens as a result of a mutation in human hemoglobin, whose intracellular concentration in human red blood cells is exceptionally high — on the order of 330 mg/mL (Krueger and Nossal 1988). Similarly, some types of cataracts are associated with mutations in *γ*S-crystallin, whose concentration in the eye lens is higher than 400 mg/mL (Brubaker et al. 2011). Consistently, highly expressed proteins evolve slowly (Yang et al. 2012), and their surfaces are subjected to negative design — show less sticky surfaces — (Levy et al. 2012) presumably to avoid misinteractions.

The risk of agglomeration is not restricted to highly expressed proteins, though. On the one hand, point mutations, especially when occurring in oligomers, can drive protein self-assembly at low concentrations (Garcia-Seisdedos et al. 2017). On the other hand, proteins have different self-assembly potentials (Garcia Seisdedos et al. 2022), due to factors such as symmetry and surface chemistry, and so, the concentration threshold at which different proteins agglomerate is different. While insolubility is suggested to shape the expression levels of proteins in the cell (Tartaglia et al. 2007), the intracellular concentration of most proteins seems to be close to or even above their solubility threshold (Tartaglia et al. 2007; Vecchi et al. 2020). Therefore, we could expect agglomeration events to be common in proteomes.

### Cellular environment

As cells are dynamic entities, so are the biophysical properties of their interiors, which cells often regulate or harness to adapt to changing environments (Garcia-Seisdedos et al. 2020; Villegas et al. 2022). For example, stress and nutrient depletion are often met with massive intracellular agglomeration events and reorganization of molecules (Minsky et al. 2002; Munder et al. 2016). Such changes in the assembly state of molecules might be provoked by changes in pH (Munder et al. 2016; Petrovska et al. 2014), macromolecular crowding (Delarue et al. 2018), solvent properties (Patel et al. 2017), and viscosity (Persson et al. 2020). Here, we comment on how these biophysical properties can affect the agglomeration of proteins (Fig. 3b–e).

#### Changes in pH

The effects of pH on protein solubility have been known for decades. In his book, *Physical Chemistry of Macromolecules*, Charles Tanford noted that to a first approximation, protein solubility is proportional to the square of its net charge (Tanford 1961). Thus, when the pH of a solution comes near the isoelectric point of a protein — the pH value at which the net charge is zero — the solubility of a protein drops to its minimum (Tanford 1961). Under these conditions, proteins display low net charge and are subject to weaker repulsive interactions, making attractive interactions dominant (Fig. 3b). Therefore, provided that their concentration is high enough, proteins can self-assemble into agglomerates (Munder et al. 2016).

While the effect the pH on the *in vitro* agglomeration of proteins was known for decades (Boye et al. 1996; Matsudomi et al. 1991; Parker et al. 2005; Renard and Lefebvre 1992), only some decades later was reported the influence of pH on protein agglomeration in a cellular context (Munder et al. 2016; Nüske et al. 2020; Petrovska et al. 2014), possibly due to the erroneous assumption that cytosolic pH was invariable in cells (Petrovska et al. 2014). Petrovska and co-workers showed that the enzyme glutamine synthase forms filaments in yeast upon acidification of the cytosol (Petrovska et al. 2014). Later, the same research group found that low pH promoted a general liquid-to-solid transition of the yeast cytosol that includes the agglomeration of many metabolic enzymes (Munder et al. 2016) and translation factors (Nüske et al. 2020). Notably, changes in pH also trigger the formation of stress granules through liquid-liquid phase separation in yeast (Riback et al. 2017).

A drop in the intracellular pH may trigger the neutralization of charges of glutamic and aspartic residues of proteins’ surfaces, decreasing the repulsive interactions and promoting the agglomeration of some proteins. Indeed, the neutralization of a few charged residues is sufficient to trigger agglomeration in many proteins (Garcia Seisdedos et al. 2022). As intracellular acidification is common upon energy depletion and other stress conditions (Orij et al. 2012; Pintsch et al. 2001; Riback et al. 2017; Weitzel et al. 1985), and negatively charged proteins with acidic pI dominate in the cytosol across organisms (Schwartz et al. 2001), acidic-driven assembly of proteins may be a general adaptive mechanism to stress (Munder et al. 2016).

#### Macromolecular crowding

In starved cells, macromolecular motility is severely restricted (Joyner et al. 2016; Parry et al. 2014). Joyner and co-workers found that such low motility could not be explained by pH changes or low energy levels but rather by increased macromolecular crowding due to water loss and cell size reduction (Joyner et al. 2016). In addition, vacuole enlargement has also been proposed to play a role in the increase of molecular crowding in starved yeast cells (Marini et al. 2020). Macromolecular crowding is also directly regulated by the central growth regulator mTORC1 by tuning the concentration of ribosomes in the cell, through production and autophagy (Delarue et al. 2018). Altogether these studies suggest that cells closely regulate the level of crowding in order to control intracellular diffusion and macromolecular interactions (Joyner et al. 2016).

Macromolecular crowding refers to the effects of adding macromolecules to a solution, as compared to a solution without any macromolecules (Aumiller et al. 2014). It is crucial for the functioning of biological systems, as crowding agents in high concentrations entropically favor the association of molecules, accelerating chemical reactions (Zhou et al. 2008). However, excessive crowding dramatically decreases molecular motility (Miermont et al. 2013; Trappe et al. 2001). The impact of crowding on molecules depends on their molecule size; molecules with large sizes are usually more affected than smaller particles (Zimmerman and Minton 1993).

In the crowded environment of the cell, protein-protein interactions are favored, as the excluded volume around the protein complex is smaller than the excluded volume of each monomeric protein unit (Minton 2000; Ralston 1990). Since agglomerates are large oligomers, crowding is expected to have a major impact on protein agglomeration (Minton 2000; Ross and Minton 1979). Indeed, excluded volume theory predicts that the addition of an inert macromolecule can significantly reduce the solubility of a protein that is in equilibrium with a condensed phase, hence promoting the formation of agglomerates and fibrillar assemblies (Ross and Minton 1979) (Fig. 3c). Such theoretical predictions have been experimentally validated. Notably, the addition of inert proteins to solutions of hemoglobin S substantially promotes and accelerates the formation of hemoglobin fibers (Behe and Englander 1978). The addition of crowding agents also lowers the critical concentration and accelerates the formation of actin and tubulin microtubules (Drenckhahn and Pollard 1986; Herzog and Weber 1978; Lindner and Ralston 1997; Tellam et al. 1983; Woodruff et al. 2017), and fibrin clot formation (Wilf et al. 1985).

More recently, Petrovska and co-workers showed that the yeast enzyme glutamine synthase needs a crowding agent at concentrations that resemble the physiological crowding of the cell to reconstitute *in vitro* filaments observed in starved cells (Petrovska et al. 2014). In consonance, the same research group showed that, in addition to pH, crowding also influences the assembly of a translation initiation factor that agglomerates in starved yeast cells (Nüske et al. 2020). These studies suggest that both pH and macromolecular crowding have strong effects on protein solubility, and they are known driving forces for the formation of agglomerates.

#### Viscosity

It was recently shown by Person and co-workers that cells regulate the synthesis of trehalose and glycogen to adjust the intracellular viscosity (Persson et al. 2020). This mechanism seems to aid yeast cells in adapting to temperature and low energy level changes by maintaining invariant diffusion rates at different temperatures. Beyond yeast cells, viscosity is tuned to promote an intracellular “glassy” state with low metabolic activity in bacteria (Parry et al. 2014), is increased in plant spores, and linked to low metabolic mobility and metabolic inactivity (Buitink and Leprince 2004), and intriguingly changes in viscosity have been observed in cancer cells (Guyer and Claus 1942; Rousset et al. 1981; Takahashi et al. 1999; Wu et al. 2019).

The variation of intracellular concentrations of trehalose and glycogen that modulate the cell’s viscosity seems to affect protein solubility and phase separation (Persson et al. 2020). Although the effect of viscosity on protein agglomeration has not been well characterized, Person and co-workers’ results suggest that high levels of glycogen and trehalose could decrease protein solubility. Suggesting that viscosity could likely modulate the agglomeration of proteins (Fig. 3d).

#### Solvent properties

The burial of hydrophobic patches at the surface of proteins is the main contributor to protein-protein interactions (Chothia and Janin 1975), which leads to the release of water molecules in bulk and an increase in entropy. The entropy gained by water compensates for the entropy lost by protein molecules forming a complex. Electrostatic interactions also contribute to the stability of protein complexes and confer interaction specificity (Chothia and Janin 1975; Derewenda 2004; Doye et al. 2004). They require surface complementarity and incorrect associations are heavily penalized by unfavorable enthalpies due to poor packing and loss of hydrogen bonds made to water (Chothia and Janin 1975). Thus, the presence of solutes that change the solvent structure, like hexanediol, modulate the strength of hydrophobic interactions, as well as the concentration of ions impacts the interactions between charged residues (Villegas et al. 2022).

The exploration of the impact of ions on protein interactions dates back more than a century to the work of Franz Hofmeister (Hofmeister 1888). He conducted studies on the salt effects on protein precipitation, particularly focusing on hen egg white proteins. In this context, protein precipitation is caused by salt-mediated protein agglomeration. He found that the ability of salts to precipitate proteins, known as “salting-out,” depended on the hydration properties of the ions. This led to the development of the Hofmeister series, an empirical ranking of both cations and anions based on their effectiveness in precipitating proteins.

These series are divided into anionic and cationic, with anionic exhibiting a stronger effect (Marcus 2009). The series are CO_3_^2−^ > SO_4_^2−^ > H_2_PO^4−^ > F^−^ > Cl^−^ > Br^−^ > NO_3_^−^ > ClO_4_^−^ > SCN^−^, and NH_4_^+^ > Cs^+^ > K^+^ > Na^+^ > Li^+^ > Ca^2+^ > Mg^2+^ > Zn^2+^, for anions and cations, respectively. Ions are ranked from kosmotropes, also termed “water structure makers,” with strong hydration causing an increase in protein stability and a decrease in protein solubility (salting-out) to chaotropes or “water structure breakers,” that increase protein solubility (salting-in) with a decrease in conformational stability (Hatefi and Hanstein 1969; Marcus 2009; Zhao et al. 2006) (Fig. 3e). Although the implications of these effects are wide in several scientific areas, we still lack a complete mechanistic understanding (Gibb 2019).

Divalent cations also play a role in modulating protein-protein interactions (Arakawa and Timasheff 1984). Their influence on the surface chemistry of proteins can lead to agglomeration, where these cations act as bridging molecules between protomers (see the “Protein surface chemistry” section). In addition, agglomeration can also be mediated via other surface-binding molecules. Those molecules may serve as bridges between protomers bringing their interaction. Bridging molecules can be DNA (Mou et al. 2015), cycles (Alex et al. 2019), heme (Kitagishi et al. 2007), biotin (Burazerovic et al. 2007; Müller et al. 2011; Petkau-Milroy et al. 2013), sugars (Hoeg-Jensen et al. 2005; Sukegawa et al. 2017), and other small molecules (Słabicki et al. 2020).

## Agglomeration in evolution

Creating novel protein interfaces leading to agglomerates might create new protein functionalities (Song and Tezcan 2014) and provide enzymatic regulation opportunities (Hvorecny and Kollman 2023). The agglomerating state might lock proteins in conformations, inactivating (Barry et al. 2014; Petrovska et al. 2014) or promoting enzymatic activities (Lynch et al. 2017), and even switching between activities in moonlighting proteins (Moon et al. 2005a). Furthermore, agglomeration might cause difficult substrate accessibility, which can be harnessed to increase substrate selectivity (Kim et al. 2005), and might boost enzymatic cooperation, facilitating substrate channeling (Chang et al. 2015). We recommend referring to Park and Horton’s review (Park and Horton 2019) for an exhaustive review of protein filamentation. We also recommend referring to recent reviews tackling how agglomeration can regulate enzymatic activity (Garcia-Seisdedos et al. 2019; Hvorecny and Kollman 2023; Liu 2016; Lynch et al. 2020; Montrose et al. 2020). Here, we give a brief overview of how agglomeration regulates the activity of some enzymes and how agglomeration can promote cellular dormancy (Montrose et al. 2020; Munder et al. 2016).

### Enzymatic regulation

Protein agglomeration can act as a mechanism of allosteric control, either by stabilizing the active or inactive states of an enzyme or by influencing the affinity for other allosteric effectors (Hvorecny and Kollman 2023). A key advantage of agglomeration in enzymatic regulation is that it is a fast and energy-efficient mechanism compared to transcription, post-translational modifications, and enzymatic degradation.

**Fig. 4:**
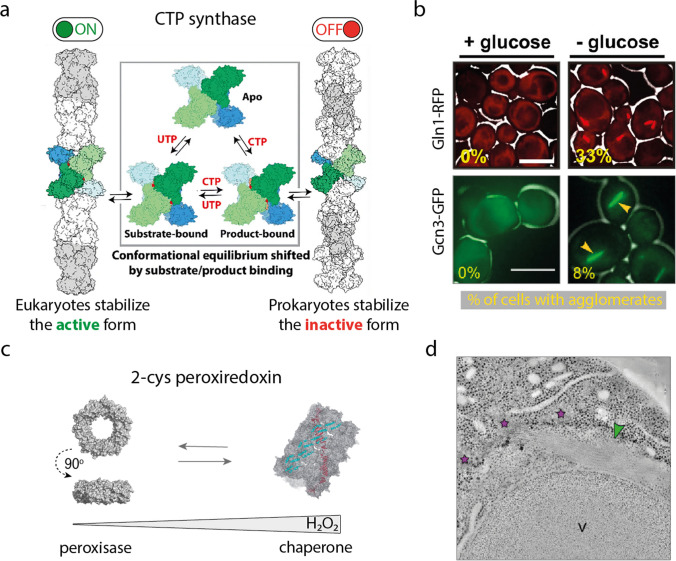
**Agglomeration in evolution**. **a** Model of allosteric regulation for human and *E. coli* CTPS. Although filament formation is present in both organisms, it has opposite effects on the enzyme activity (adapted with permission of Lynch et al. (2017)). **b** In *S. cerevisiae*, the enzyme glutamine synthase (Gln1) and the transcription initiation factor eIF2B (Gcn3) form filaments upon glucose deprivation (adapted from Petrovska et al. 2014, and Nüske et al. 2020). **c** Hydrogen peroxide–induced polymerization of the enzyme 2-cys peroxiredoxin serves as a functional switch from peroxidase to chaperone activity (adapted with permission from Saccoccia et al. 2012). **d** TEM image displaying eIF2B filaments in a starved yeast cell (image reproduced from Marini et al. 2020)

Agglomeration frequently inhibits enzymatic activity. In the case of *E. coli* CTP synthase, filamentation stabilizes the inactive conformation of CTP protomers and, as a consequence, inhibits CTP activity (Fig. 4a) (Barry et al. 2014). Yeast Glucokinase 1 (Glk1), a key enzyme that regulates entry into glycolysis, is active as a monomer, and it is typically expressed when glucose levels are low. When glucose levels spike, Glk1 assembles into filaments that inactivate its activity by preventing substrate turnover (Stoddard et al. 2020). Also, in yeast, glutamine synthase (Gln1), an essential metabolic enzyme catalyzing the synthesis of glutamine from glutamate and ammonium, assembles in filaments under starvation conditions inactivating its activity (Fig. 4b) (Petrovska et al. 2014). Agglomeration can also inactivate the function of other proteins, this is the case of the yeast translation initiation factor eIF2B (Gcn3), which forms inactive filaments under starvation downregulating protein translation (Fig. 4b) (Nüske et al. 2020). The target of rapamycin serine/threonine kinase (TOR) has two complexes TORC1 and TORC2 that regulate cell growth and metabolism. Recently, it was reported that under glucose deprivation, yeast TORC1 assembles into helical fibers inhibiting TOR activity (Prouteau et al. 2017).

Agglomeration can also promote enzymatic activity, as exemplified by the human CTP synthases. Filamentation of CTP synthase 1 stabilizes the active conformation increasing enzymatic activity (Fig. 4a) (Lynch et al. 2017). CTP synthase 2 forms dynamic filaments that can switch between inhibited and active states through cooperative interactions between protomers along the filament. This cooperativity enables rapid adaptation to changes in nucleotide demand in cells based on substrate and allosteric inhibitor balance (Lynch and Kollman 2020). Human glutaminase C polymerization into filaments also promotes its catalytic activity, and its allosteric inhibitor, BPTES, inhibits the enzyme by disrupting the filaments (Ferreira et al. 2013). The binding of allosteric activators to the enzyme glutamate dehydrogenase (GDH) increases its activity and induces the formation of filaments. On the other hand, the binding of inactivators decreases the enzyme’s activity and leads to the dissociation of filaments. This suggests an association between enzymatic activity and the filamentous form of GDH (Fahien et al. 1989; Frieden 1959; Gylfe 1976; Park and Horton 2019).

Other enzymes like acetyl Co-A and IMPDH can assemble into filaments that adopt both active and inactive conformations, shifting from one to the other upon binding substrates and allosteric modulators (Anthony et al. 2017; Beaty and Lane 1983; Hunkeler et al. 2018; Kim et al. 2010; Park and Horton 2019; Simonet et al. 2020).

Agglomeration can also switch activities in multifunctional proteins, that is the case of human 2-Cys peroxiredoxins, where the formation/dissociation of filaments triggers a functional switch between chaperone and peroxidase activities (Fig. 4c) (Moon et al. 2005b)

### Cellular dormancy

Recent studies have reported that key metabolic enzymes conditionally assemble into filaments in response to nutrient scarcity (Marini et al. 2020; Minsky et al. 2002; Nüske et al. 2020; Petrovska et al. 2014). Some studies propose that when certain enzymes form filaments, they deactivate their function, leading to a metabolic shutdown and putting cells in a dormant state (Marini et al. 2020; Munder et al. 2016; Nüske et al. 2020; Petrovska et al. 2014). Most of these studies have been conducted in *S. cerevisiae*, where the enzyme glutamine synthase, the translation initiation factor eIF2B, and the master regulator TORC1 have been demonstrated to downregulate their function through agglomeration triggered by nutrient deprivation (Fig. 4b and d) (Nüske et al. 2020; Petrovska et al. 2014; Prouteau et al. 2017). Notably, upon glucose addition, the assemblies dissolve and cells resume growth (Nüske et al. 2020; Petrovska et al. 2014). Thus, protein agglomeration constitutes an energy-independent and reversible mechanism for cells to shut down their metabolism, reducing costs, and entering in a growth-arrested state allowing them to survive the lack of nutrients.

Under starvation, and consequently, under low energy levels, cell interiors can undergo a liquid-to-solid transition (Minsky et al. 2002; Munder et al. 2016; Parry et al. 2014) where general assembly of proteins is promoted (Minsky et al. 2002). In fact, physical factors known to change with cellular state, like pH, crowding, or energy levels, influence intra-cellular protein assembly (Munder et al. 2016; Petrovska et al. 2014) (see section “Factors linked to agglomeration”) and phase-separation (Franzmann et al. 2018; Villegas et al. 2022). In addition to a mechanism to promote metabolic shutdown, agglomeration of proteins has been proposed to constitute storage depots (Munder et al. 2016) and to prevent damage to vital molecular components under acute stress (Minsky et al. 2002).

## Agglomeration in disease

**Fig. 5:**
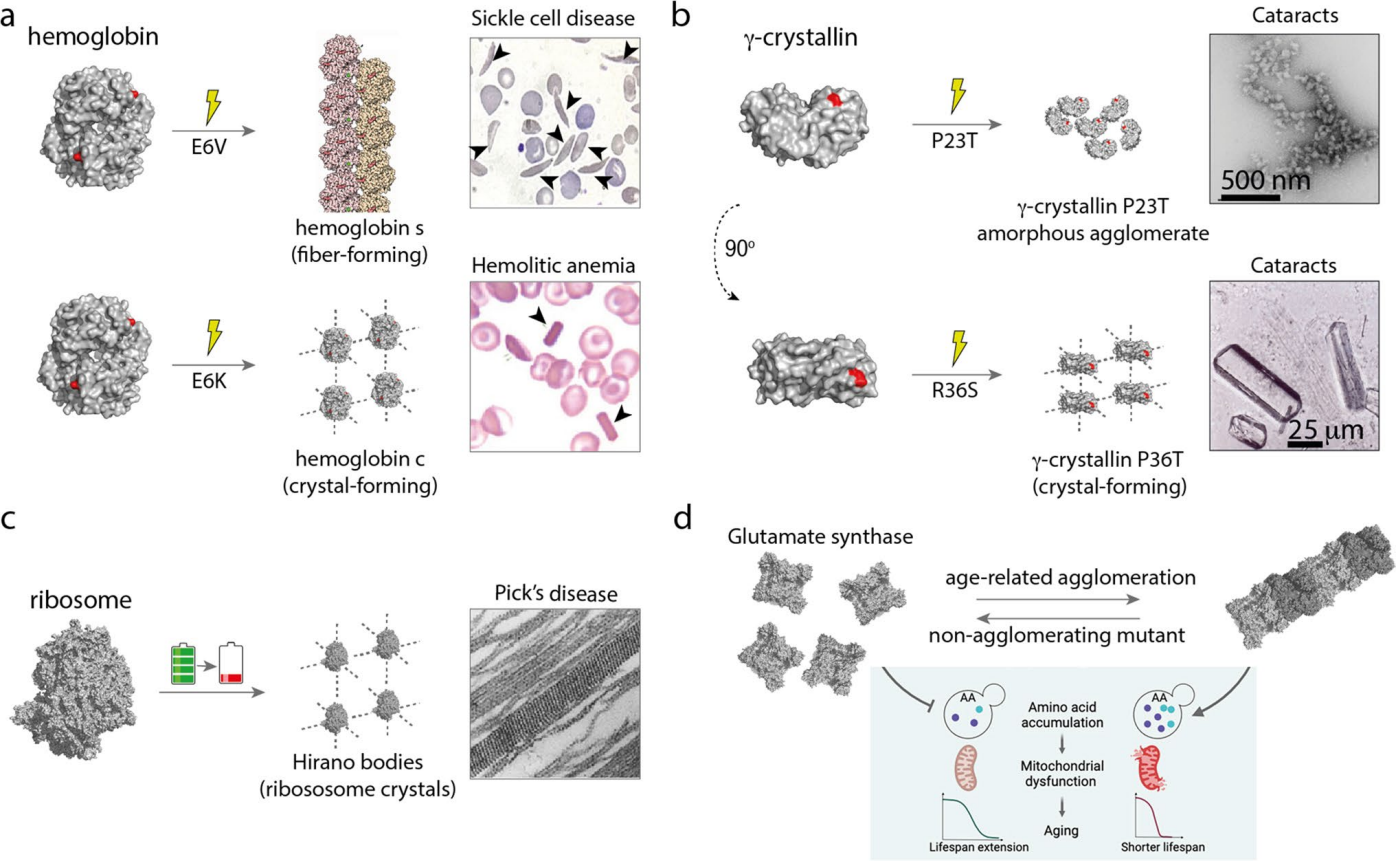
**Agglomeration in disease and aging**. **a** Mutations E6V and E6K in human hemoglobin cause sickle cell disease and hemolytic anemia respectively (images reproduced from Harrington et al. 1997, and from the ASH Image Bank — This image was originally published in ASH Image Bank. Luiz Arthur Leite, PhD; Danilo Retucci; Fabiana Conti, MD; Vinicius de Moraes. Title: Sickle cell anemia in a hemolytic crisis. ASH Image Bank. 2021; Image # 00063634. © the American Society of Hematology.). **b** Mutations P23T and P36T trigger agglomeration of *γ*-crystallin in the eye lens causing cataracts (images reproduced from Boatz et al. 2017, and Kmoch et al. 2000). **c** Ribosome crystals isolated from brain cells of a patient with Pick’s disease (image reproduced from (O’Brien et al. 1980)). **d** The metabolic enzyme glutamate synthase (gtl1) forms agglomerates in aged yeast cells triggering amino acid accumulation and mitochondrial dysfunction that results in a shorter lifespan compared to non-agglomerating mutants of gtl1 (image modified with permission of Paukštytė et al. 2023)

The fact that point mutations can frequently cause proteins to form agglomerates, as it has been recently demonstrated (Garcia-Seisdedos et al. 2017; Garcia Seisdedos et al. 2022), suggests that agglomeration could be related to many disease mutations. Perhaps the most paradigmatic case of agglomeration in disease is that of hemoglobin in sickle cell disease (Eaton and Hofrichter 1990), whereby the mutation E6V at the surface of human hemoglobin triggers the formation of filaments causing the red blood cells to acquire their characteristic sickle shape (Fig. 5a). Notably, the filaments form in the de-oxygenated form of the hemoglobin and dissolve upon re-oxygenation exhibiting the characteristic reversible nature of agglomerates. A less famous disease occurring also in hemoglobin is caused by a mutation in the same residue but to a lysine. The hemoglobin mutant E6K agglomerates form intracellular crystals causing hemolytic anemia (Fig. 5a) (Charache et al. 1967; Vekilov et al. 2002). The assemblies are also reversible, but opposite to in sickle cell disease, they form in the oxygenated form and dissolve upon de-oxygenation.

Other diseases associated with agglomeration include cataracts, where some mutants in γD-crystallin form large assemblies of folded proteins in the eye lens scattering light and causing vision impairment (Fig. 5b) (Boatz et al. 2017; Kmoch et al. 2000; Pande et al. 2005). In addition, agglomeration appears to be linked to some mutant forms of SOD1 in ALS (Garcia-Seisdedos et al. 2019; Pratt et al. 2014). Changes in filament assembly parameters of the enzyme IMPDH induced by mutations are linked to rhinitis pigmentosa (Burrell et al. 2022; Labesse et al. 2013; Thomas et al. 2012). Intriguingly, large crystalline assemblies of ribosomes, known as Hirano bodies, can be found in brain cells of aged senile humans, and are a histological feature in brain tissue of patients afflicted with neurodegenerative diseases like Pick’s disease, Alzheimer’s disease, and Creutzfeld-Jacob’s disease (Fig. 5c) (Minsky et al. 2002; O’Brien et al. 1980).

### Agglomeration in aging

Recently, Paukštytė and co-workers found that glutamate synthase (Glt1) polymerizes into agglomerates during aging in *S. cerevisiae*, causing the breakdown of cellular amino acid homeostasis. Notably, they found that inhibiting Glt1 polymerization by mutating the polymerization interface restored amino acid levels in aged cells, attenuating mitochondrial dysfunction, and promoting lifespan extension (Fig. 5d) (Paukštytė et al. 2023).

## Concluding remarks

Here, we have focused on the phenomenon by which proteins form open-ended assemblies in their folded state. A phenomenon that we termed agglomeration to differentiate it from other open-ended assembly phenomena with similar macroscopic features such as aggregation. We reviewed the main structural, chemical, and physical factors linked to protein agglomeration, as well as some of the implications of agglomeration in health, disease, and aging. We hope this review will contribute to raising awareness within the scientific community regarding the prevalence and significance of this phenomenon. We aim to inspire researchers to actively explore and characterize any potential instances of agglomeration they may come across.
